# Pharmacologic targeting of Nedd8-activating enzyme reinvigorates T-cell responses in lymphoid neoplasia

**DOI:** 10.1038/s41375-023-01889-x

**Published:** 2023-04-08

**Authors:** Xiaoguang Wang, Canping Chen, Dan Vuong, Sonia Rodriguez-Rodriguez, Vi Lam, Carly Roleder, Jing H. Wang, Swetha Kambhampati Thiruvengadam, Allison Berger, Nathan Pennock, Pallawi Torka, Francisco Hernandez-Ilizaliturri, Tanya Siddiqi, Lili Wang, Zheng Xia, Alexey V. Danilov

**Affiliations:** 1 City of Hope National Medical Center, Duarte, CA USA; 2 Computational Biology Program, Department of Biomedical Engineering, Oregon Health & Science University, Portland, OR USA; 3 Department of Medicine, Division of Hematology/Oncology, University of Pittsburgh, Pittsburgh, PA USA; 4 Takeda Development Center Americas, Cambridge, MA USA; 5 Knight Cancer Institute, Oregon Health & Science University, Portland, OR USA; 6 Division of Hematology & Medical Oncology, Roswell Park Cancer Institute, Buffalo, NY USA

**Keywords:** Translational research, B-cell lymphoma

## Abstract

Neddylation is a sequential enzyme-based process which regulates the function of E3 Cullin-RING ligase (CRL) and thus degradation of substrate proteins. Here we show that CD8^+^ T cells are a direct target for therapeutically relevant anti-lymphoma activity of pevonedistat, a Nedd8-activating enzyme (NAE) inhibitor. Pevonedistat-treated patient-derived CD8^+^ T cells upregulated TNFα and IFNγ and exhibited enhanced cytotoxicity. Pevonedistat induced CD8^+^ T-cell inflamed microenvironment and delayed tumor progression in A20 syngeneic lymphoma model. This anti-tumor effect lessened when CD8^+^ T cells lost the ability to engage tumors through MHC class I interactions, achieved either through CD8^+^ T-cell depletion or genetic knockout of *B2M*. Meanwhile, loss of *UBE2M* in tumor did not alter efficacy of pevonedistat. Concurrent blockade of NAE and PD-1 led to enhanced tumor immune infiltration, T-cell activation and chemokine expression and synergistically restricted tumor growth. shRNA-mediated knockdown of HIF-1α, a CRL substrate, abrogated the in vitro effects of pevonedistat, suggesting that NAE inhibition modulates T-cell function in HIF-1α-dependent manner. scRNA-Seq-based clinical analyses in lymphoma patients receiving pevonedistat therapy demonstrated upregulation of interferon response signatures in immune cells. Thus, targeting NAE enhances the inflammatory T-cell state, providing rationale for checkpoint blockade-based combination therapy.

## Introduction

Immunotherapy revolutionized cancer care: immune checkpoint inhibitors received FDA approvals for treatment of multiple tumor types, and cell-based therapies (i.e., chimeric antigen receptor T cells) transformed standard of care of patients with non-Hodgkin lymphoma (NHL) and acute lymphoblastic leukemia. More recently, bi-specific T-cell engagers have shown impressive clinical activity in hematologic malignancies [1]. While such strategies are highly effective, they remain non-curative and novel approaches to enhance their clinical impact are necessary.

Contrary to chemotherapy, novel targeted therapies are associated with diverse immunomodulatory effects [2, 3]. For example, inhibitors of B-cell receptor-associated kinases, now widely used in therapy of NHL, may enhance T-cell function through multiple mechanisms, including reversal of exhaustion, altered T-cell polarization, and neutralization of the immunosuppressive effect by the neoplastic cells [3, 4]. Importantly, the clinical efficacy of compounds which target protein degradation, such as the E3 ubiquitin ligase modulator lenalidomide, in large part depends on their immunomodulatory effects [5, 6]. Thus, investigation of pathways within the ubiquitin-proteasome system (UPS) which may be engaged to enhance anti-tumor immunity is of critical importance.

Cullin-RING E3 ubiquitin ligases (CRLs) conjugate ubiquitin chains to unwanted or misfolded proteins in cells, eventually leading to proteasome-mediated degradation [7]. To undergo the conformational change and enable substrate ubiquitination, the CRLs require conjugation of the active ubiquitin-like protein NEDD8 to the scaffold Cullin proteins, a process referred to as neddylation [8]. Like ubiquitination, neddylation is a three-step sequential enzymatic reaction which starts with NEDD8 activation by the E1 NEDD8-activating enzyme (NAE, NAE1/UBA3 heterodimer), followed by NEDD8 transfer to the E2 NEDD8-conjugating enzymes (UBC12 and UBE2F) and finally NEDD8 addition to the Cullin proteins [9].

Pevonedistat (MLN4924, TAK924) is a small molecule that covalently adducts with NEDD8, rendering it inactive and eventually leading to CRL deactivation and accumulation of CRL-dependent substrates [10]. Targeting neddylation with pevonedistat demonstrated preclinical and early clinical efficacy across a spectrum of hematologic malignancies and solid tumor types, alone and in combination with chemotherapeutic, hormonal and targeted agents [10–20]. On the other hand, neddylation has been implicated in regulation of immune cell function [21–23]. Our group first demonstrated that pharmacologic targeting NAE rehabilitated T cells derived from patients with chronic lymphocytic leukemia (CLL), leading to decreased Treg differentiation and enhanced T_H1_ polarization, accompanied by increased production of interferon-γ (IFN-γ), and confirmed these findings in immunized mice [21]. However, whether altering neddylation may ultimately enhance anti-tumor immunity, and contribute to the direct anti-tumor effects of pevonedistat in lymphoma and other cancers, remains unknown. In the current work, we employed the A20 syngeneic lymphoma mouse model and primary samples from patients treated with pevonedistat to demonstrate that targeting neddylation directly enhances anti-tumor response by promoting T-cell activation.

## Methods

### Patient samples

Following approval by the Institutional Review Board and provision of written informed consent, peripheral blood was obtained from CLL patients treated at City of Hope National Medical Center (Duarte, CA). Peripheral blood samples were obtained from eight patients with NHL and CLL who received pevonedistat on a Phase I clinical trial (at baseline, 3 and 24 h after pevonedistat infusion; Supplementary Table S1; NCT03479268) [24]. Peripheral blood mononuclear cells (PBMCs) were isolated using standard Ficoll-Hypaque technique (Amersham). Red blood cells were lysed using ACK buffer (Thermo Fisher Scientific).

### Cell culture

A20, JeKo-1, Mino, Val, Maver-1 and Lewis lung carcinoma (LLC) cells were obtained from American Type Culture Collection (ATCC, USA). Mycoplasma testing was performed regularly (every 2 months) using Mycoplasma PCR detection kit (ABM, Canada). The number of passages between thawing and use in the described experiments ranged between two and five. Cells were cultured in RPMI-1640 medium supplemented with 15% fetal bovine serum, 100 U/mL penicillin, 100 μg/mL streptomycin, 2 mM l-glutamine, 25 mM HEPES, 100 μM nonessential amino acids, and 1 mM sodium pyruvate (Lonza).

Total CD3^+^ T cells were isolated from PBMCs derived from CLL patients by positive selection kit (Invitrogen, USA) and cultured with 0.5 μg/mL plate-bound anti-CD3 (clone UCHT1) and 0.5 μg/mL soluble anti-CD28 (clone CD28.2; BD Biosciences).

Pevonedistat (TAK924) was provided by Takeda Development Center Americas, Inc.

### Cell viability testing

To measure cell proliferation, cells were plated in 96-wellplates (5000 cells/well, 3 wells/sample) with drugs and incubated for 48 h at 37 °C in 5% CO_2_. After incubation, relative numbers of viable cells were measured using a tetrazolium-based colorimetric assay (CellTiter Aqueous One Solution Cell Proliferation Assay, Promega).

For cytotoxicity assay, stimulated T cells were harvested and washed three times with PBS. Lymphoma cells were labeled with CFSE and co-cultured with T cells in 1:1 or 5:1 ratio for additional 48 h. Apoptosis was measured using the ApoScreen Annexin V Apoptosis Kit (Southern Biotech). Briefly, cells were resuspended in 100 µL Annexin V binding buffer containing 0.5 µL Annexin V mAb and 0.5 µL 7-aminoactinomycin D (7-AAD). Cells were then subjected to flow cytometry and double-positive (Annexin V/&-AAD) population was analyzed.

### Immunoblotting

Cells were lysed in RIPA buffer (20 mM Tris, 150 mM NaCl, 1 % NP-40, 1 mM NaF, 1 mM sodium phosphate, 1 mM NaVO_3_, 1 mM EDTA, 1 mM EGTA), supplemented with protease inhibitor cocktail (Roche), phosphatase inhibitor cocktail 2 (Sigma-Aldrich) and 1 mM phenylmethanesulfonyl fluoride. Primary antibodies used in Western blot analysis were listed in Supplementary Table S2. Secondary horseradish peroxidase-conjugated anti-mouse and anti-rabbit were purchased from Cell Signaling Technology. Protein bands were developed by ECL Western blotting detection reagents (GE Healthcare) according to kit instructions.

### In vivo experiments

Six-to-eight-week-old mice were purchased from The Jackson Laboratory (United States). Mice were injected subcutaneously at both flanks with 1 × 10^6^ A20 lymphoma cells (WT BALB/c) or LLC cells (WT C57BL/6 (B6)). When tumors reached 100 mm^3^, tumor-bearing recipient mice were randomized into groups and treated as indicated. All mice were maintained under specific pathogen-free conditions in the vivarium facility of City of Hope. Animal work was approved by the Institutional Animal Care and Use Committee of City of Hope. The following drugs were used: InVivoMAb anti-mouse CD8α (clone 53-6.7), InVivoPlus anti-mouse PD-1 (clone RMP1-14; both from BioXCell).

### Tumor dissociation and flow cytometry

Tumors were harvested from tumor-bearing mice. Tumor weight was measured before dissociation and tumors were processed into single-cell suspension. Antibodies used for flow cytometry were listed in Supplementary Table S2. For tumors and human PBMC cells, dead cells were excluded by Live/Dead fixable aqua dead cell stain kit (Invitrogen). BD Fix/Permeabilization buffer was used for intracellular staining of IFN-γ, TNF-α, IL-2, IL-4 and IL-17 in tumor-infiltrating lymphocytes (TILs).

For cytokine staining, equal numbers of tumor (or human PBMC cells) were cultured in vitro for 5 h in the presence of 50 ng/mL phorbol 12-myristate 13-acetate (PMA) (Sigma Aldrich), 1 μg/mL ionomycin (Sigma Aldrich) and 5 μg/mL BFA (Biolegend). For FoxP3 staining, True-nuclear Transcription Factor Buffer Set was used (Biolegned). Data were acquired on BD Fortessa and analyzed with FlowJo software V10 (Oregon, USA).

### Statistical analysis

At least 3 biological replicates were used in all experiments shown throughout the manuscript, unless noted otherwise. Statistical analysis was performed with Student t test or one-way ANOVA with Tukey’s multiple comparisons test, when indicated, in GraphPad Prism software. *p < 0.05, **p < 0.01 and ***p < 0.001 throughout the manuscript. Research reported in this publication included work performed in the Hematopoietic Tissue Biorepository, the Integrative Genomics and Bioinformatics Core and the Analytical Cytometry Cores and supported by the National Cancer Institute of the National Institutes of Health under award number P30CA033572.

## Results

### Pharmacologic targeting NAE enhances T-cell cytotoxicity in vitro

**Fig. 1:**
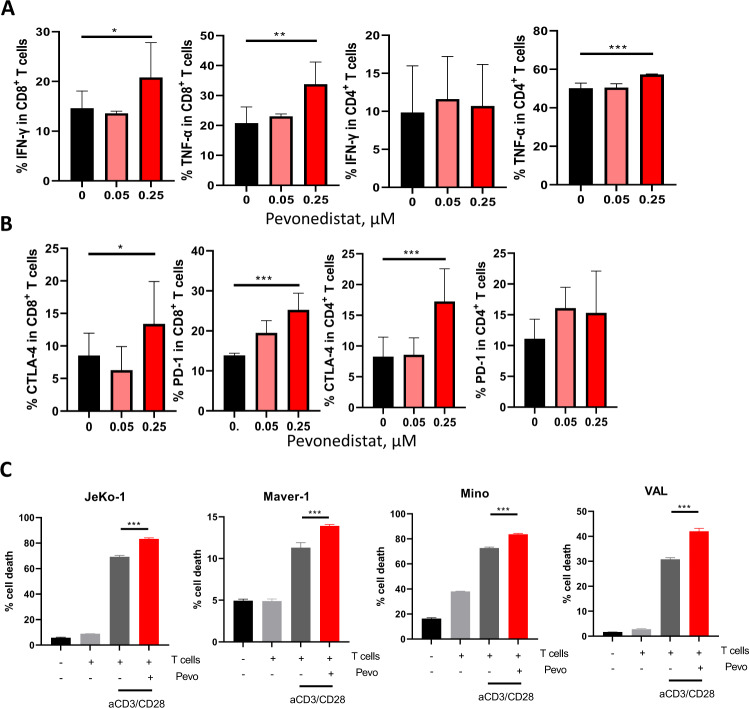
NAEi modulates CD8^+^ T-cell response. Magnetically enriched CD3^+^ cells derived from patients with CLL (*n* = 3 individual patients) were activated with 0.5 μg/mL αCD3/28 for 24 h. Thereafter, stimulation continued in the presence of the indicated doses of pevonedistat or vehicle control for an additional 72 h. **A**, **B** Expression of IFNγ, TNFα, CTLA-4 and PD-1 was quantified in CD4^+^ or CD8^+^ T cells by flow cytometry. **C** T cells were treated as previously, followed by pevonedistat wash off. NHL cells were labeled with CFSE and co-cultured with T cells for additional 48 h. Cell death was quantified using Annexin V/7-AAD expression by flow cytometry. Data are mean ± SD. One-way ANOVA was performed for statistical analysis, *p < 0.05, **p < 0.01, ***p < 0.001.

We have previously shown that NAE inhibition-induced IFN-γ and downmodulated IL-2 expression in primary CD4^+^ T cells [21]. To investigate whether interference with the neddylation pathway altered the functionality of cytotoxic lymphocytes, primary T cells isolated from peripheral blood of CLL patients were subjected to TCR engagement followed by treatment with pevonedistat. We observed that NAE inhibition led to increased secretion of the effector molecules TNF-α and IFN-γ in human CD8^+^ T cells (Fig. 1A; Supplementary Fig. 1A). By contrast, TNF-α expression and IFN-γ by CD4^+^ T cells was unchanged. Microarray analysis of sorted naïve CD3^+^ T cells has previously demonstrated mRNA transcript upregulation of the exhaustion markers following pevonedistat treatment [21]. Here we observed that CTLA-4 expression was induced by pevonedistat in both CD8^+^ and CD4^+^ T cells, while only CD8^+^ T cells exhibited upregulation of PD-1 (Fig. 1B). Next, we evaluated the effect of pevonedistat on T-cell mediated cytotoxicity. While unstimulated patient-derived T cells failed to exhibit cytotoxicity, CD3/28 stimulation led to a significant killing of target lymphoma cells (Fig. 1C; Supplementary Fig. 1B). Pre-treatment with pevonedistat enhanced allogeneic T-cell-mediated killing of neoplastic B cells, suggesting that NAE inhibition may enhance cytotoxic cell function.

### NAE inhibition activates tumor-infiltrating lymphocytes (TILs)

**Fig. 2:**
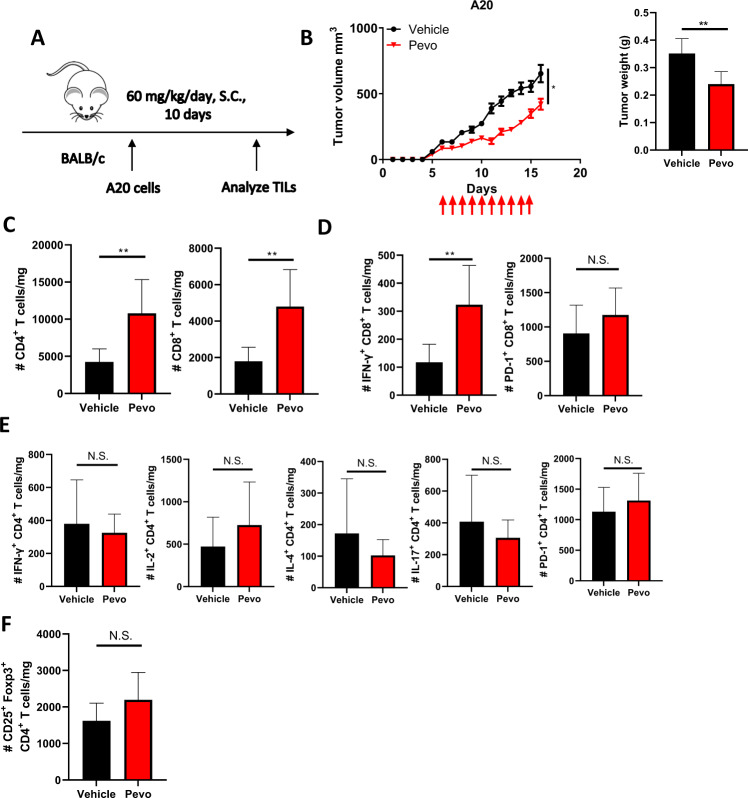
Pevonedistat inhibits A20 tumor progression and modulates TILs. **A** A20 lymphoma cells were transplanted subcutaneously into syngeneic recipient mice. Mice were treated with pevonedistat 60 mg/kg for 10 days or vehicle control (*n* = 6 per group). Tumors were harvested on day 16 after inoculation and subsequently used for TIL analysis. **B** Tumors were measured every other day; tumor volumes at day 16 are shown on the right. **C**–**F** Immune profiles of CD4^+^ and CD8^+^ TILs assayed at the end of the experiment. Data are mean ± SD. One-way ANOVA was performed for statistical analysis, *p < 0.05, **p < 0.01.

We had shown that NAE inhibition results in increased IFN-γ secretion and a reduction in *T*_reg_ population in ova immunization model, suggesting that NAE inhibition may modulate anti-tumor response in vivo [21]. To further explore the immunomodulatory effect of NAE inhibition in an immunocompetent model, A20 lymphoma cells were transplanted into syngeneic BALB/c mice. Tumors emerged approximately one week after inoculation. When tumor size reached ~100 mm^3^, mice were randomized into two groups and treated with either pevonedistat or vehicle control for 10 days (Fig. 2A). In this model, treatment with pevonedistat restricted tumor growth (Fig. 2B). Analysis of murine tumors at the end of treatment revealed that the absolute quantities of CD4^+^ and CD8^+^ TILs were significantly increased by pevonedistat (Fig. 2C; Supplementary Fig. 2). Furthermore, we observed an increased number of IFN-γ-producing CD8^+^ cells in tumors harvested from pevonedistat-treated mice compared with control animals (Fig. 2D). Meanwhile, treatment with pevonedistat had no effect on cytokine secretion by CD4^+^ cells (Fig. 2E). Expression of PD-1 was also unchanged in both T-cell subsets (Fig. 2D, E). Additionally, we did not observe a change in the infiltrating Treg cells (Fig. 2F).

To explore whether immunomodulation via NAE inhibition was restricted to the highly immunogenic A20 lymphoma model, we conducted experiments in Lewis lung carcinoma (LLC) which is considered a relatively immunologically cold tumor. Still, treatment with pevonedistat restricted growth of LLC tumors (Supplementary Fig. 3A). Interestingly, here we also observed a dramatic increase in both CD4^+^ and CD8^+^ TILs, albeit the absolute lymphocyte numbers were significantly lower than in the A20 model (Supplementary Fig. 3B). Furthermore, unlike in A20 model, LLC TILs exhibited an increased expression of immune checkpoint molecules upon NAE inhibition (Supplementary Fig. 3C).

Thus, NAE inhibition modulated TILs in syngeneic mouse models.

### CD8^+^ T cells are indispensable for the therapeutic effect of pevonedistat

**Fig. 3:**
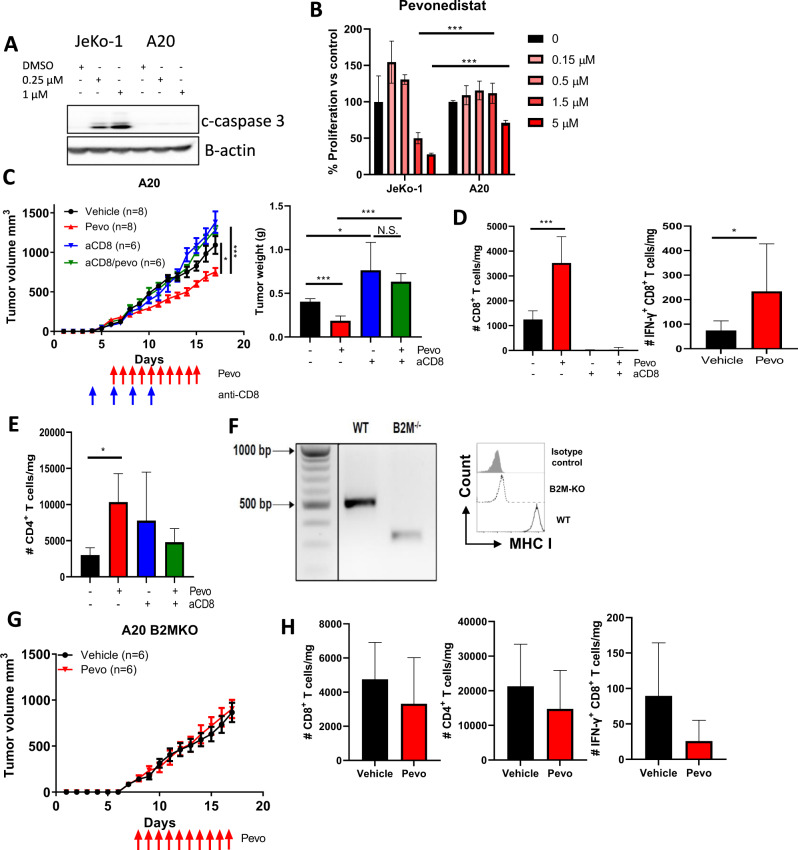
Therapeutic effect of pevonedistat depends on CD8^+^ T cells. **A** A20 cells were treated with the indicated concentrations of pevonedistat for 24 h. Total protein lysates were subjected to immunoblotting. JeKo-1 cells were used as a positive control. **B** JeKo-1 or A20 cells were treated with the indicated doses of pevonedistat for 48 h. Cell proliferation was measured by colorimetric MTS assay. % of viable cells were calculated against untreated controls. **C** A20 lymphoma cells were transplanted into syngeneic recipient mice. Mice were treated with 60 mg/kg pevonedistat or vehicle control for 10 days with or without an anti-CD8 antibody (250 μg i.p. on days 4, 6, 8, and 10 after tumor inoculation). Tumors were measured at the end of the experiment and used for TIL analysis. **D**, **E** Immune profiles of tumor-infiltrating CD8^+^ and CD4^+^ T cells. **F** PCR products and a representative flow cytometry histogram of WT vs *B2M*^−/−^ A20 cells are shown. **G** A20 *B2M*-KO mice were treated with pevonedistat versus vehicle control as previously (*n* = 6 each). Tumor growth curve is shown. **H** Immune profiles of CD8^+^ and CD4^+^ TILs are shown. Data are mean ± SD. One-way ANOVA was performed for statistical analysis, *p < 0.05, ***p < 0.001.

We and others have shown that pevonedistat exerts direct anti-tumor effects across multiple tumor types [14, 16, 25–27]. Still, our findings strongly suggest that immune modulation significantly contributes to the therapeutic effect of pevonedistat. To further explore this, we first evaluated susceptibility of A20 cells to NAE inhibition in vitro. While pevonedistat is cytotoxic to multiple NHL cell lines, A20 cells were resistant to NAE inhibition (Fig. 3A, B) [26, 27]. To investigate the relative importance of TILs in pevonedistat-mediated anti-tumor effect, we used antibody-mediated depletion of CD8^+^ T cells in mice. Interestingly, loss of CD8^+^ T cells resulted in accelerated tumor growth, further implicating the immune environment in this mouse model (Fig. 3C). The therapeutic effect of NAE inhibition was also completely abrogated in lymphoma-bearing mice treated with anti-CD8 antibody. Consistent with our earlier data, the absolute numbers of both CD8^+^ TILs and IFN-γ-producing CD8^+^ cells were significantly increased by pevonedistat treatment, while expression of PD-1 remained unchanged (Fig. 3D**;** Supplemental Fig. 4A). As expected, CD8^+^ TILs were not detected in mice treated with anti-CD8 antibody, while the phenotypic and compositional profiles of the CD4^+^ T cells were unaffected (Fig. 3D, E; Supplementary Fig. 4B).

Since cytotoxic T cells require MHC class I molecules for antigen presentation, we explored whether their loss impacts pevonedistat efficacy. We had previously employed the CRISPR/Cas9 technique to generate knockout of β2-microglobulin (*B2M*-KO) [28]. The *B2M* knockout completely abolished the expression of MHC class I in A20 cells (Fig. 3F). While B2M loss did not negatively impact tumor growth either in vitro or in vivo, the anti-tumor effect of pevonedistat was fully abrogated (Fig. 3G). *B2M*-KO tumors did not attract TILs whose profiles from pevonedistat-treated mice were comparable with control animals (Fig. 3H; Supplementary Fig. 3C, D).

**Fig. 4:**
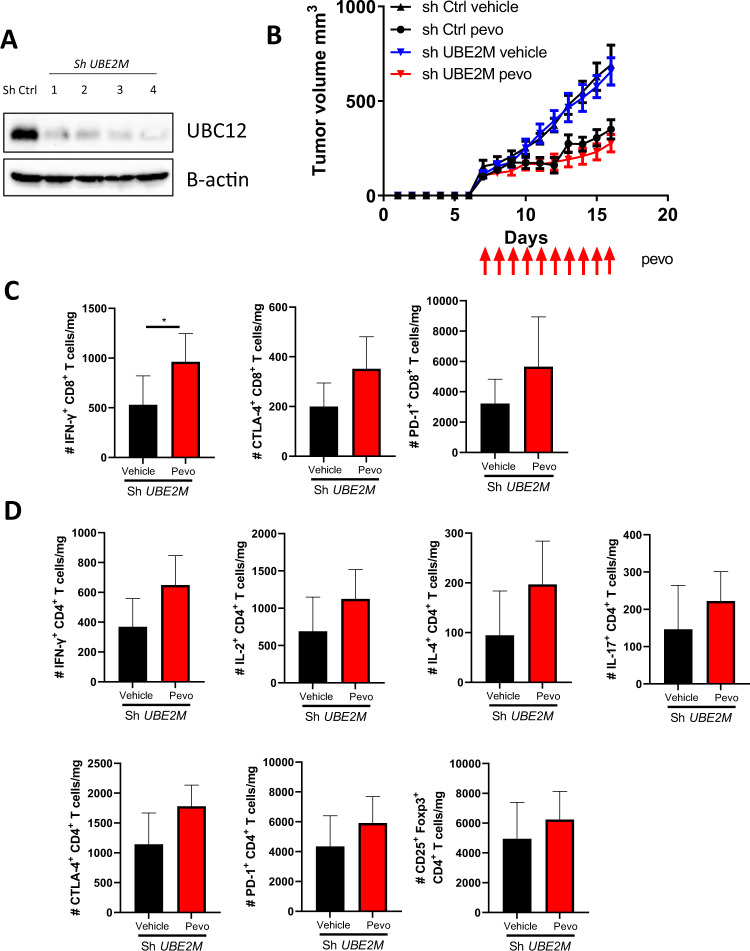
Therapeutic effect of pevonedistat does not depend on tumor-intrinsic effects. A20 cells were transduced with lentivirus bearing *UBE2M* shRNA and stable knockdown cell lines were selected by puromycin. sh*UBE2M* or control A20 cells were transplanted into syngeneic recipient mice, tumor-bearing mice were randomized into 2 groups and treated with vehicle control or pevonedistat 60 mg/kg for 10 days. **A** Whole-cell lysate proteins from A20 tumors collected at the end of mouse experiment were subjected to immunoblotting. 4 individually obtained cell lines are shown. **B** Tumor growth of A20 lymphoma upon pevonedistat treatment (*n* = 6). **C**, **D** Immune profiles of CD8^+^ and CD4^+^ TILs. Data are mean ± SD. *p < 0.05.

To further investigate the exact contribution of NAE inhibition in the tumor, we employed shRNA-mediated knockdown of *UBE2M* in A20 cells. UBC12, encoded by *UBE2M*, is NEDD8-conjugating enzyme which governs neddylation [23]. sh*UBE2M* A20 cells were transplanted into flanks of syngeneic BALB/c mice followed by pevonedistat treatment. We confirmed durability of UBC12 knockdown in transplanted A20 tumors (Fig. 4A). Surprisingly, we found that the therapeutic effect of pevonedistat was not altered by the intratumoral loss of UBC12 (Fig. 4B). Furthermore, TILs from pevonedistat-treated mice whose tumors lacked UBC12 exhibited the same functional features as TILs in control mice (Fig. 4C, D; Supplementary Fig. 5).

Taken together, our data strongly implicate CD8^+^ T cells as a mediator of the anti-tumor effect of NAE inhibition.

### HIF1A mediates NAE inhibition-induced anti-tumor immunity

**Fig. 5:**
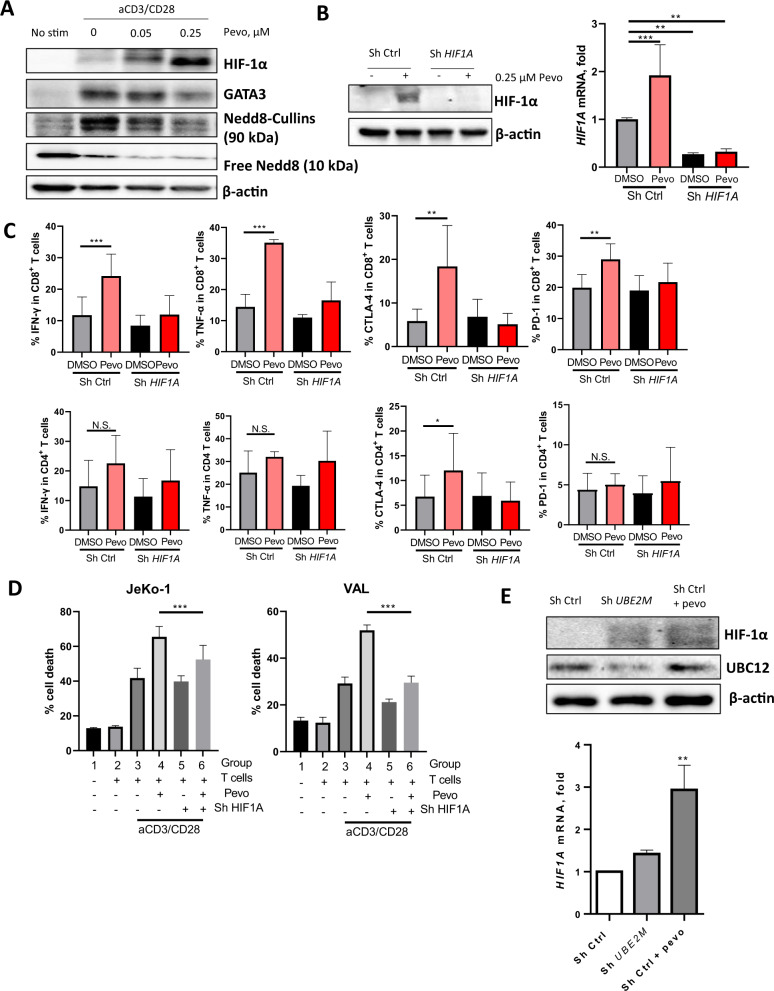
HIF-1α mediates NAE effect in T cells. Magnetically enriched CD3^+^ cells were activated with 0.5 μg/mL αCD3/28 for 24 h. **A** Thereafter, stimulation continued in the presence of the indicated doses of pevonedistat or vehicle control for an additional 72 h. Protein expression was quantified by immunoblotting. **B** T cells were transduced by lentivirus bearing with *HIF1A* shRNA (or vector control) overnight. Thereafter, stimulation continued in the presence of 0.25 μM pevonedistat or vehicle control for an additional 72 h. HIF-1α expression was quantified by immunoblotting (left) and RT-PCR (right). **C** Expression of IFNγ, TNFα, CTLA-4 and PD-1 was quantified in CD8^+^ T cells by flow cytometry. **D** T cells were stimulated with CD3/28 and treated with pevonedistat as above. The drug was washed off, and T cells were co-cultured with CFSE-labeled JeKo-1 or VAL cells for 48 h. Apoptosis was quantified by flow cytometry using Annexin V/7-AAD. Data are mean ± SD. One-way ANOVA was performed for statistical analysis, **p < 0.01, ***p < 0.001. **E** T cells were transduced with *UBE2M* shRNA (or vector control). Thereafter, stimulation continued in the presence of 0.25 μM pevonedistat or vehicle control for an additional 72 h. HIF-1α expression was quantified by immunoblotting and RT-PCR.

The mechanism of neddylation-mediated immune regulation is understudied. CRL substrates which accumulate following blockade of NAE enzymatic activity may mediate immune effects. Among these substrates, GATA3 and HIF-1α have been shown to modulate expression of immune checkpoint molecules and cytokine secretion by immune cells [29–31]. Hence, they were further investigated here. Treatment with pevonedistat led to accumulation of HIF-1α in TCR-stimulated primary patient-derived T cells in vitro (Fig. 5A). By contrast, we observed a dose-dependent downregulation of GATA3 following pevonedistat exposure of human T cells. We next generated *HIF1A* knockdown in primary patient-derived T cells (Fig. 5B). Unlike control cells, CD4^+^/CD8^+^ human T cells which lacked HIF-1α failed to upregulate checkpoint molecules and secrete cytokines upon treatment with pevonedistat in vitro (Fig. 5C, Supplementary Fig. 6). Furthermore, loss of HIF-1α partially abrogated NAE inhibition-induced cytotoxicity of allogeneic T cells (Fig. 5D), suggesting that immunomodulatory effect of pevonedistat is at least in part mediated via HIF-1α.

To further elucidate the role of CRL blockade in HIF-1α upregulation, we generated *UBE2M* knockdown in primary patient-derived T cells (Fig. 5E). As predicted, *UBE2M* knockdown resulted in upregulation of HIF-1α protein expression, albeit to a lesser degree than pevonedistat treatment. This was likely explained by incomplete knockdown of *UBE2M*. Interestingly, we also observed that treatment with pevonedistat increased HIF-1α mRNA transcript levels (Fig. 5B), suggesting that pevonedistat mediates HIF-1α levels via additional mechanisms, not limited to regulation of protein turnover.

### NAE inhibition enhances efficacy of PD-1 blockade

**Fig. 6:**
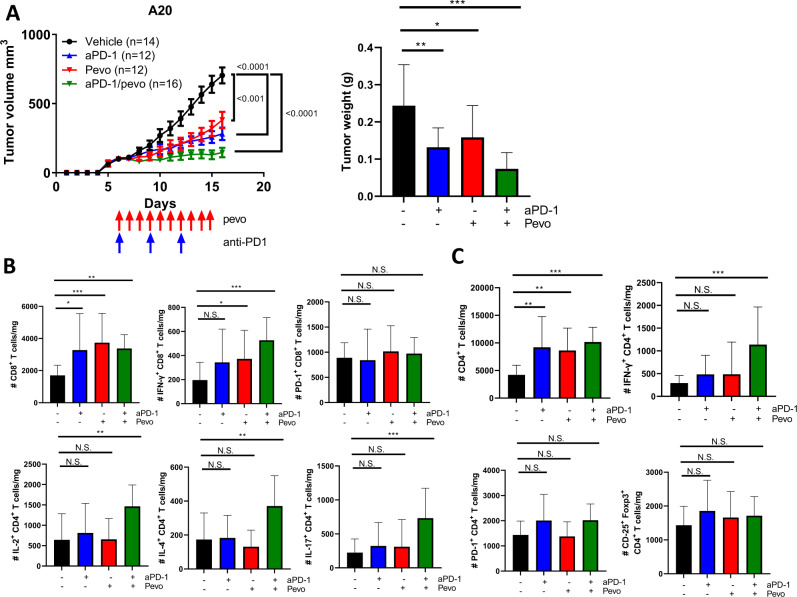
Combined targeting NAE and PD-1 in A20 lymphoma. **A**–**C** A20 lymphoma cells were transplanted into syngeneic recipient mice, and tumor-bearing mice were randomized into 4 groups and treated with vehicle control, pevonedistat (as previously), anti-PD-1 antibody (200 μg/mouse/dose, i.p., on days 6, 9, and 12), or both. Tumors were measured daily and at the end of the experiment (right panel) and subsequently were used for TIL analysis. Immune profiles of CD4^+^ and CD8^+^ TILs are shown. *p < 0.05, **p < 0.01, ***p < 0.001.

We postulated that since NAE inhibition enhances T-cell anti-tumor immunity, pevonedistat may sensitize lymphoid tumors to immune checkpoint inhibitors. While PD-1 inhibition exhibited a partial anti-tumor effect, combined treatment with pevonedistat arrested in vivo growth of A20 lymphomas (Fig. 6A). This was accompanied by increased IFN-γ production in both CD8^+^ and CD4^+^ murine TILs (Fig. 6B, C). Moreover, combined treatment upregulated secretion of multiple cytokines by the CD4^+^ TILs (Fig. 6C). As expected, the combination of pevonedistat and anti-PD-1 had no therapeutic efficacy in A20 *B2M*-KO lymphomas (Supplementary Fig. 7A, C).

Previous studies have shown that pevonedistat may upregulate PD-L1 expression by the tumor cells via MYC oncogene [18, 32]. Consistent with this notion, we found that treatment with pevonedistat upregulated MYC expression, but not IFN-γ, in A20 cells in vitro (Supplementary Fig. 7D, E). Furthermore, both in vitro and in vivo treatment with pevonedistat led to increased PD-L1 expression by A20 cells (Supplementary Fig. 7F), possibly accounting for their sensitization to combined PD-1 blockade. Coupled with the above evidence that inactivation of the neddylation pathway in the tumor does not negative impact in vivo effect of pevonedistat, this supports the idea that immune effects of NAE inhibition likely result from a direct effect on T cells.

### Immunomodulatory effect of pevonedistat patients with NHL

**Fig. 7:**
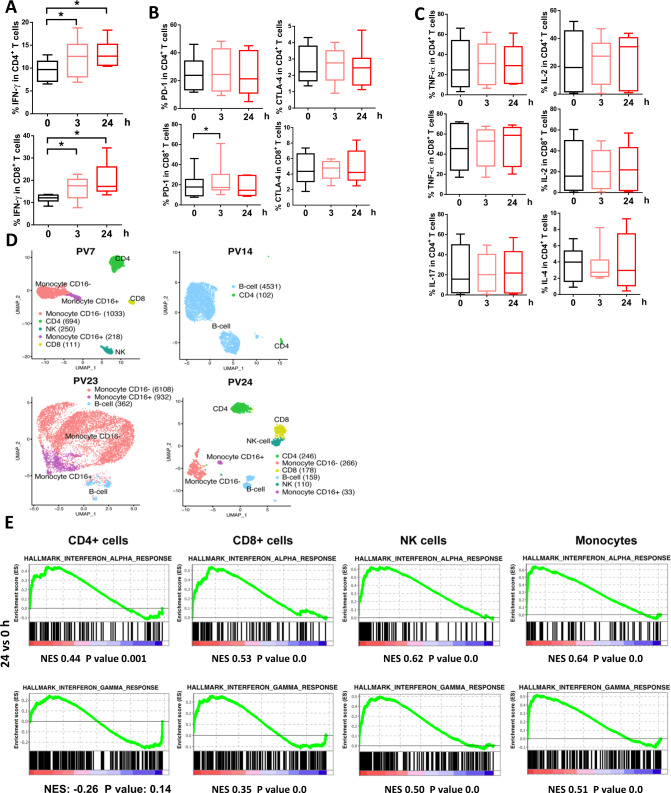
Clinical effect of pevonedistat in NHL patients. PBMC were collected at baseline and 3 and 24 h post-pevonedistat infusion. For cytokine staining, equal numbers of human PBMC cells were cultured in vitro for 5 h in the presence of PMA, ionomycin and BFA. **A** IFN-γ **B** CTLA-4 and PD-1 and **C** TNFα, IL-2, IL-4 and IL-17 expression was quantified in CD4^+^ or CD8^+^ T cells by flow cytometry. Data are mean ± SEM, *p < 0.05. **D** UMAP (uniform manifold approximation and projection) visualization of all cells colored by cell type. **E** GSEA (gene set enrichment analysis) plots illustrating the significant enrichment of hallmark IFN-α and IFN-γ response pathways in 24 h post infusion versus baseline in the indicated cell types. The green line represented the enrichment of pathway in the 24 h post infusion group or control baseline group according to gene expression levels, with genes enriched in the 24 h post infusion group shown on the left and genes enriched in the control baseline group shown on the right. NES normalized enrichment score.

To evaluate relevance of our findings to human disease, we analyzed T cells from patients with NHL who received pevonedistat on the Phase I clinical trial (NCT03479268) [24]. PBMCs were isolated from blood samples collected at baseline (prior to pevonedistat first dose), and 3 and 24 h after pevonedistat infusion. We observed enhanced IFN-γ secretion in CD4^+^ and CD8^+^ T cells as early as 3 h post infusion (Fig. 7A). The induction of IFN-γ in CD8^+^ T cells persisted for at least 24 h. Meanwhile, expression of either PD-1 or CTLA-4 was not affected by pevonedistat (Fig. 7B). As expected, treatment with pevonedistat had no immediate effect on production of other cytokines (Fig. 7C).

Additionally, paired samples from 4 patients with NHL enrolled on the above study were submitted for scRNA-Seq to study the effect of pevonedistat on immune cells. After quality control 2306 (PV7), 4533 (PV14), 7402 (PV23) and 992 cells (PV24) were obtained. In the published analysis, we documented rapid downregulation of NFκB signaling in malignant B cells following pevonedistat infusion [24]. Here we conducted immune cell population analysis. Between 2 and 6 immune cell types were identified in each patient, including CD4^+^, CD8^+^ T cells, CD16^+^ and CD16^-^ monocytes, B cells and NK cells (Fig. 7D). For each immune cell type, we examined the effect of pevonedistat on the transcriptome across multiple patients. Pathway level analysis confirmed downregulation of “TNFα signaling via NFκB” in CD8^+^ T cells, CD16^+^ monocytes and NK cells 24 h post infusion, consistent with our earlier above and the established target of NAE inhibition (data not shown). Meanwhile, “IFN-α response” was upregulated in CD4^+^ and CD8^+^ T cells as early as after 3 h of pevonedistat infusion (Supplementary Fig. 8A). Furthermore, upregulation of IFN-α and IFN-γ response was observed 24 h post infusion in T cells, monocytes and NK cells (Fig. 7E), indicating that pevonedistat triggered interferon signaling and thus reinvigorating T cells responses in vivo. Consistent with known mechanistic effects of pevonedistat, we detected activation of DNA repair pathways across multiple cell types (Supplementary Fig. 8B). We also detected upregulation of HIF-1α gene signatures in CD4^+^ T cells (Supplementary Fig. 8C).

Taken together, this data indicate that pharmacologic targeting NAE had a distinct immunomodulatory effect in vivo.

## Discussion

Here we demonstrate that selective NAEi enhances anti-tumor immunity in vitro and in syngeneic lymphoma mouse models. We have previously shown that pevonedistat downmodulated IL-2 production and blunted proliferation of the CD4^+^ T cells derived from patients with CLL [21]. Current in vitro analysis uncovered additional effects, demonstrating upregulation of IFN-γ and TNF-α by the TCR-stimulated CD8^+^ cells treated with pevonedistat. Consistent with earlier data, both T-cell subsets showed increased expression of CTLA-4, a checkpoint inhibitor, which could be related to NAEi-mediated downregulation of NFAT and FoxP3 [21, 33, 34].

Importantly, we observed increased in vitro cytotoxic effects of the NAEi-conditioned T cells against the lymphoma cell lines, prompting us to investigate pevonedistat effects in vivo. Treatment with pevonedistat resulted in delayed progression of A20 lymphoma tumors, associated with increased CD4^+^ and CD8^+^ T-cell infiltration. Furthermore, we have shown that the therapeutic effect of pevonedistat was T-cell-dependent, since (1) depletion of CD8^+^ T cells and (2) genetic knockout of β2-microglobulin in A20 lymphoma cells fully reversed the anti-tumor effect. Emerging data suggest that neddylation is capable of regulating the tumor microenvironment, including macrophages, dendritic cells, NK cells and T cells [21–23, 35–39].

The evidence of the role of neddylation in T-cell biology remains limited and controversial. Loss of the Nedd8-conjugating enzyme UBC12 in CD4^+^ T cells led to diminished proliferation, skewed T_H1_/T_H2_ differentiation and reduced cytokine production [36]. Pevonedistat had comparable effects in a malaria murine model, where deficiency of *UBA3*, which encodes the catalytic subunit of the NAE, significantly compromised survival, activation, and proliferation of CD4^+^ T cells and impaired T_H1_/T_FH_ differentiation [37]. Furthermore, pevonedistat was shown to suppress the ability of dendritic cells to stimulate murine and human allogeneic T-cell responses [40]. However, despite the data regarding the role of neddylation in non-cancer settings, an emerging body of data favors the notion that targeting NAE may ultimately enhance T/NK cell immunity. A recent study has shown that pevonedistat may limit tumor infiltration by the immune suppressor cells, including tumor-associated macrophages and myeloid derived suppressor cells, and promote CD8^+^ T-cell infiltration in lung cancer models [41]. In multiple myeloma models, NAE inhibition upregulated NKG2D ligands, leading to the activation of NK cells [22]. Here we provide evidence that neddylation critically restraints anti-tumor immunity, while its inhibition reverses this effect. This effect is not limited to A20 model, but also occurs in the immunologically “cold” Lewis lung carcinoma. Therefore, it is likely applicable across a spectrum of tumor tissues.

In this study, we observed significant effects of pevonedistat on CD8^+^ T cells. Yet we did not fully elucidate contribution of other cells to anti-tumor immunity. In fact, given our earlier work demonstrating that NAE inhibition suppressed expansion of Treg cells and increased CD4^+^ T-cell polarization towards T_H1_ phenotype in vitro and in ova-stimulation model in vivo [21], CD4^+^ T cells likely significantly contribute to anti-tumor effect. Ongoing studies should further clarify the interplay between the immune cell fractions upon NAE inhibition.

We further show that pevonedistat cooperates with checkpoint inhibition in the A20 model. Our result is consistent with published reports. As an example, concurrent targeting of NAE and PD-L1 restored anti-tumor immunity in prostate cancer models [42]. Genetic or pharmacologic targeting neddylation has previously been shown to upregulate PD-L1 expression in gliomas via MYC [18, 32]. While here we also found increased PD-L1 expression by A20 cells exposed to pevonedistat, whether the combined effect was due to inhibiting neddylation in the tumor versus a T-cell-intrinsic effect or modulation of additional immune microenvironment signaling [43], has not been addressed. To this end, McGrail et al. suggested that NAE inhibition may enhance tumor antigenicity. They reported that presence of microsatellite instability (MSI) enhanced tumor cells dependence on neddylation to clear misfolded protein aggregates resulting from destabilizing mutations, leading to immunologic cell death with co-targeting NAE and PD-1 [44]. By contrast, we for the first time demonstrate that immunologic effects of NAEi are fully T-cell-dependent. Genetic abolition of the neddylation pathway in A20 cells did not limit growth of control tumors and, more importantly, did not abolish the anti-tumor effect of pevonedistat. This gives further confidence that the immunomodulatory effect of NAEi will be conserved across multiple tumor types, irrespective of tumor genetic mutations.

The observed immunomodulatory effect of pevonedistat is not entirely surprising given its effect on E3 ligase function. In a recent study, avadomide, a Cereblon E3 ligase modulator, was shown to induce type I and II IFN signaling in T cells, reinvigorating T-cell responses in NHL models [45]. Avadomide promoted T-cell proliferation and chemokine synthesis, upregulated PD-L1 in the immune tumor microenvironment, and stimulated activation of cytotoxic CD8^+^ T cells when combined with anti-PD-L1 antibodies in patient-derived xenograft tumors. The immunomodulatory effect of lenalidomide and other drugs in this class have long been exploited in therapy of hematologic malignancies [46].

How exactly does pevonedistat achieves immunologic effects? Here we have shown that pevonedistat upregulates expression of HIF-1α, an established CRL protein substrate. In addition to that, we also observed increased *HIF1A* mRNA transcript levels in cells treated with pevonedistat. *HIF1A* knockdown compromised NAE-inhibition mediated T-cell cytotoxicity in vitro, indicating that Nedd8-HIF-1α is a critical pathway that governs immune response. HIF-1α deficiency has been shown to cause reduced activation and tumor infiltration by the CD8^+^ cytotoxic T cells, leading to compromised tumor cell killing and alteration of tumor vascularization [31]. Furthermore, checkpoint receptor expression (TIM3, PD-1, CTLA-4 and LAG-3), is in part regulated by HIF-1α [31]. Thus, upregulation of CTLA-4 by pevonedistat in T cells observed by us may also indirectly implicate HIF-1α signaling pathway as a key NAEi mediator. HIF-1α turnover is regulated by VHL and mutations in VHL, many occurring in the region interacting with CRLs, disrupt the ubiquitylation of HIF-1α [47–49]. This mechanism remains an area of active investigation by our group.

Finally, we have shown that some of the effects observed preclinically can be recapitulated in patients with NHL. We used scRNA-Seq technology to demonstrate that patients who received pevonedistat on a clinical trial exhibited disruption in NFκB signaling and DNA repair pathways, consistent with known targets of NAE inhibition [10]. And yet, a single pevonedistat infusion resulted in upregulated IFN-γ secretion by CD8^+^ T cells, as well as rapid induction of IFN signaling pathways across multiple cell types. Taken together, our preclinical and clinical data strongly suggest that neddylation is an important target which can be exploited to enhance the anti-tumor immunity. Perhaps the future studies should focus on combination strategies where pevonedistat is employed to enhance efficacy of checkpoint inhibitors, bi-specific antibodies or cellular therapies.

## Supplementary information

Supplementary Figures

Supplementary Methods

## Data Availability

The data that support the findings of this study are available from the corresponding author upon reasonable request.
