# Supplementary material for: Pharmacologic targeting of Nedd8-activating enzyme reinvigorates T-cell responses in lymphoid neoplasia
Source: Leukemia. 2023 Apr 8;37(6):1324–35. doi: 10.1038/s41375-023-01889-x (PMC10244170; doi:10.1038/s41375-023-01889-x)
Supplement: Supplementary file 1 — Supplementary Figures [file 41375_2023_1889_MOESM1_ESM.pptx]

## Slide 1
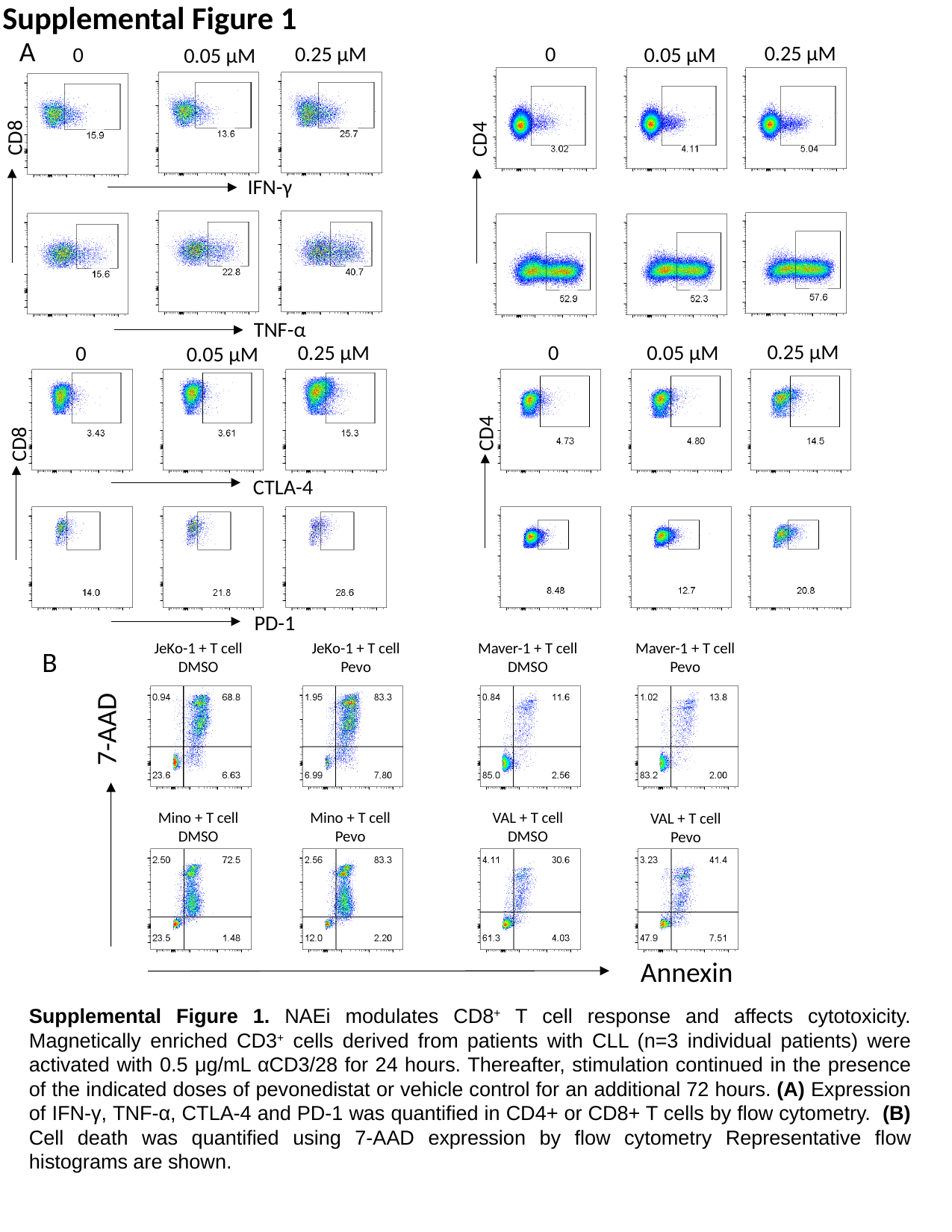

Supplemental Figure 1
A
0.25 µM
0
0.25 µM
0.05 µM
0
0.05 µM
CD8
CD4
IFN-γ
TNF-α
0.25 µM
0
0.25 µM
0.05 µM
0
0.05 µM
CD4
CD8
CTLA-4
PD-1
Maver-1 + T cell
DMSO
Maver-1 + T cell
Pevo
JeKo-1 + T cell
DMSO
JeKo-1 + T cell
Pevo
B
7-AAD
Mino + T cell
Pevo
VAL + T cell
DMSO
Mino + T cell
DMSO
VAL + T cell
Pevo
Annexin
Supplemental Figure 1. NAEi modulates CD8+ T cell response and affects cytotoxicity. Magnetically enriched CD3+ cells derived from patients with CLL (n=3 individual patients) were activated with 0.5 μg/mL αCD3/28 for 24 hours. Thereafter, stimulation continued in the presence of the indicated doses of pevonedistat or vehicle control for an additional 72 hours. (A) Expression of IFN-γ, TNF-α, CTLA-4 and PD-1 was quantified in CD4+ or CD8+ T cells by flow cytometry. (B) Cell death was quantified using 7-AAD expression by flow cytometry Representative flow histograms are shown.

## Slide 2
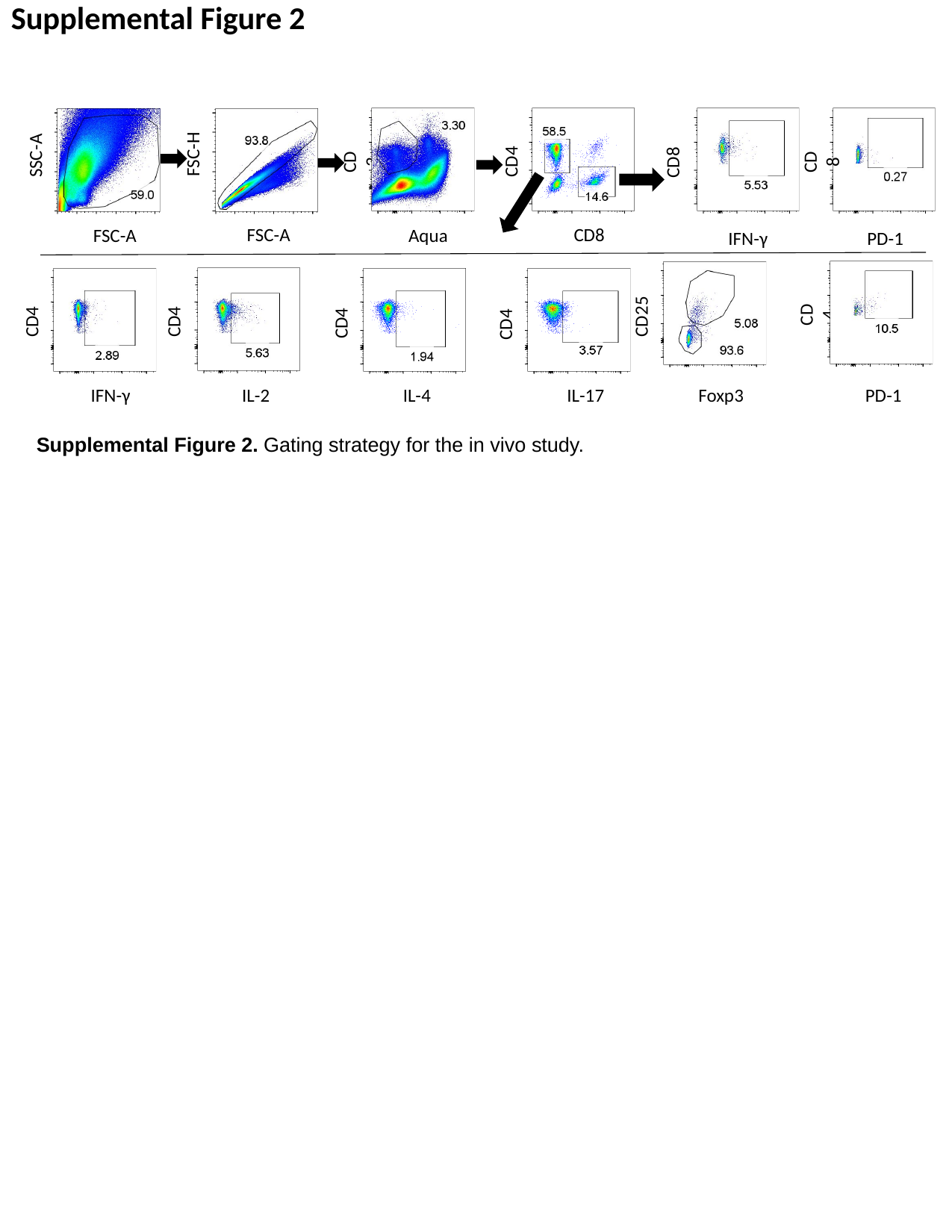

Supplemental Figure 2
SSC-A
FSC-A
FSC-H
FSC-A
CD3
Aqua
CD4
CD8
CD8
IFN-γ
CD8
PD-1
CD4
PD-1
CD25
Foxp3
CD4
IL-4
CD4
IL-17
CD4
IL-2
CD4
IFN-γ
Supplemental Figure 2. Gating strategy for the in vivo study.

## Slide 3
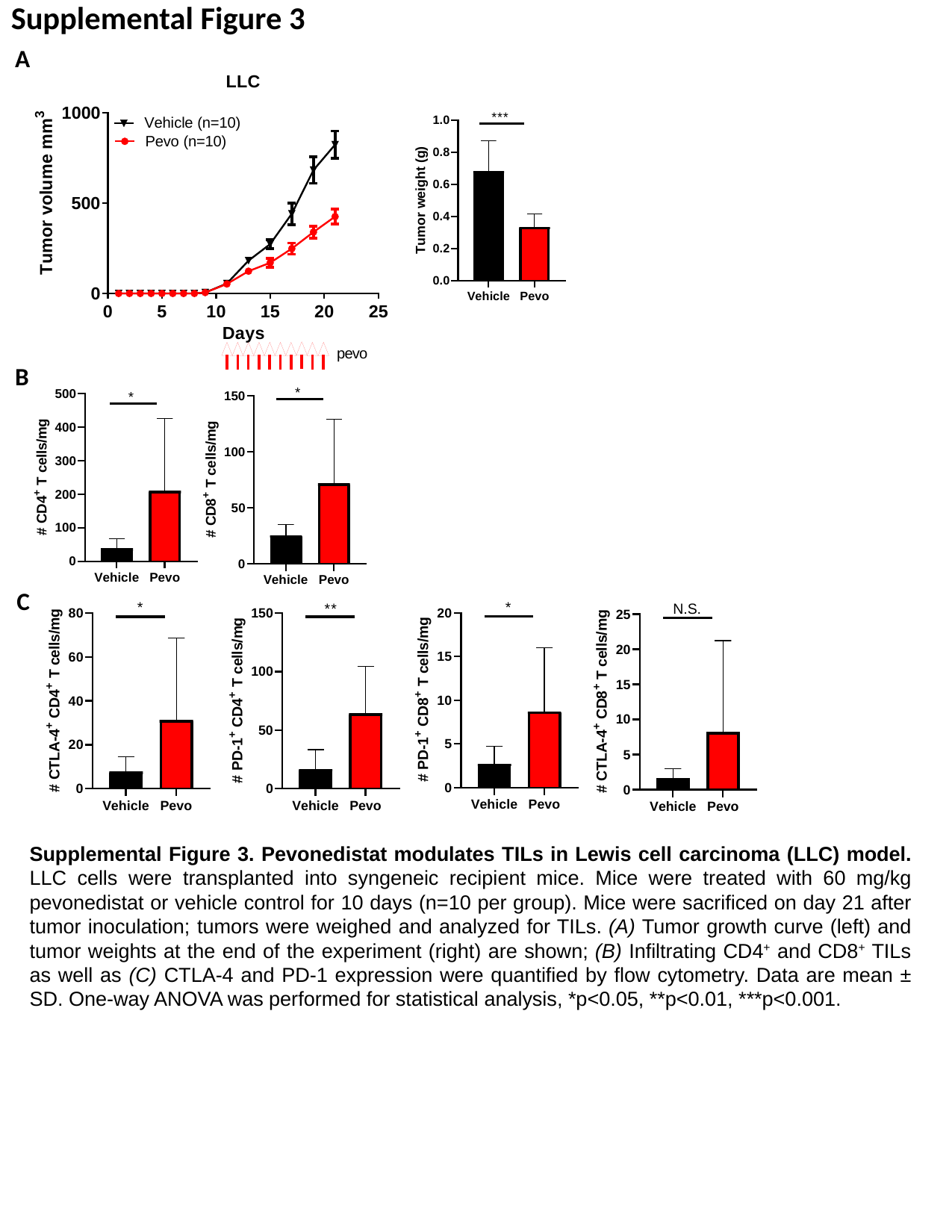

Supplemental Figure 3
A
B
C
Supplemental Figure 3. Pevonedistat modulates TILs in Lewis cell carcinoma (LLC) model. LLC cells were transplanted into syngeneic recipient mice. Mice were treated with 60 mg/kg pevonedistat or vehicle control for 10 days (n=10 per group). Mice were sacrificed on day 21 after tumor inoculation; tumors were weighed and analyzed for TILs. (A) Tumor growth curve (left) and tumor weights at the end of the experiment (right) are shown; (B) Infiltrating CD4+ and CD8+ TILs as well as (C) CTLA-4 and PD-1 expression were quantified by flow cytometry. Data are mean ± SD. One-way ANOVA was performed for statistical analysis, *p<0.05, **p<0.01, ***p<0.001.

## Slide 4
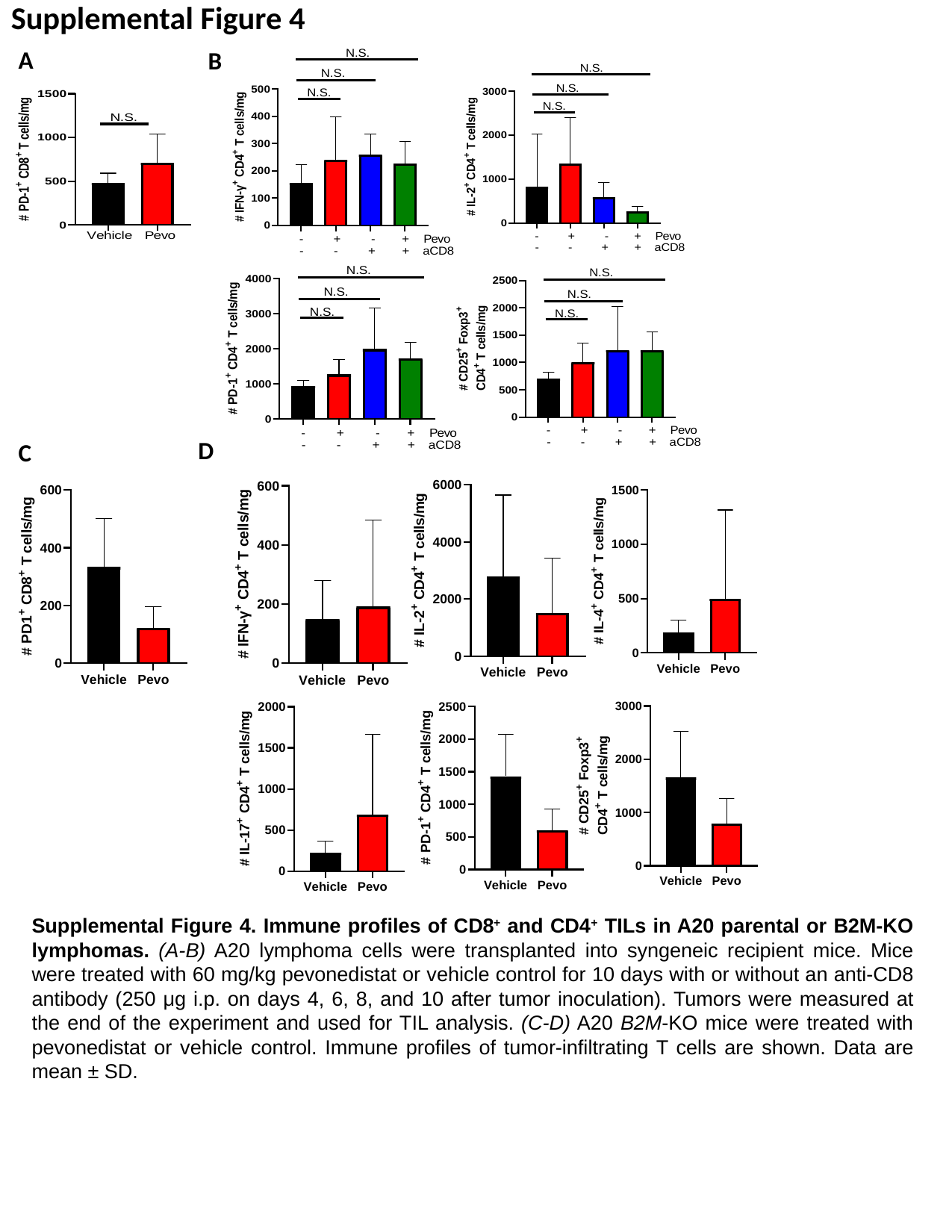

Supplemental Figure 4
A
B
D
C
Supplemental Figure 4. Immune profiles of CD8+ and CD4+ TILs in A20 parental or B2M-KO lymphomas. (A-B) A20 lymphoma cells were transplanted into syngeneic recipient mice. Mice were treated with 60 mg/kg pevonedistat or vehicle control for 10 days with or without an anti-CD8 antibody (250 μg i.p. on days 4, 6, 8, and 10 after tumor inoculation). Tumors were measured at the end of the experiment and used for TIL analysis. (C-D) A20 B2M-KO mice were treated with pevonedistat or vehicle control. Immune profiles of tumor-infiltrating T cells are shown. Data are mean ± SD.

## Slide 5
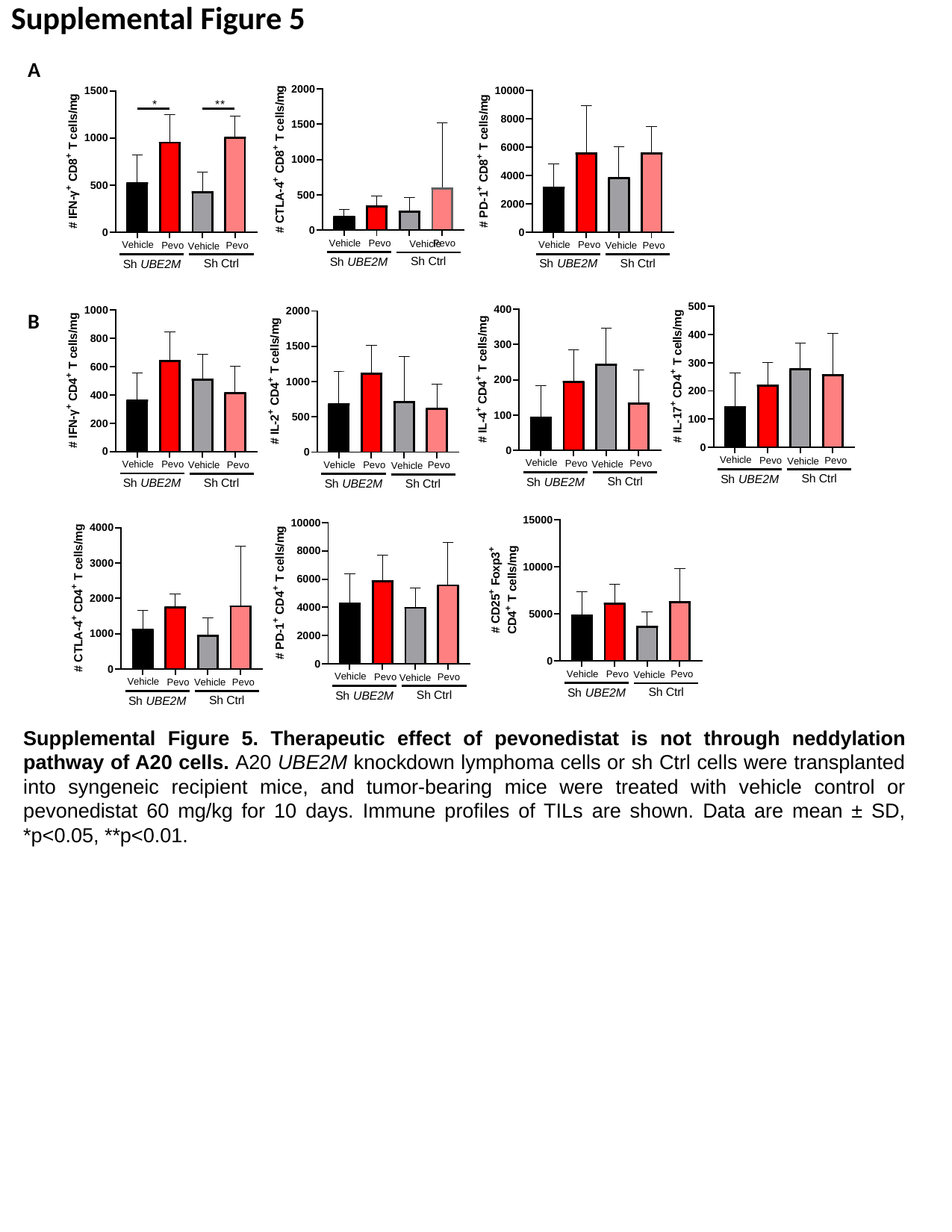

Supplemental Figure 5
A
B
Supplemental Figure 5. Therapeutic effect of pevonedistat is not through neddylation pathway of A20 cells. A20 UBE2M knockdown lymphoma cells or sh Ctrl cells were transplanted into syngeneic recipient mice, and tumor-bearing mice were treated with vehicle control or pevonedistat 60 mg/kg for 10 days. Immune profiles of TILs are shown. Data are mean ± SD, *p<0.05, **p<0.01.

## Slide 6
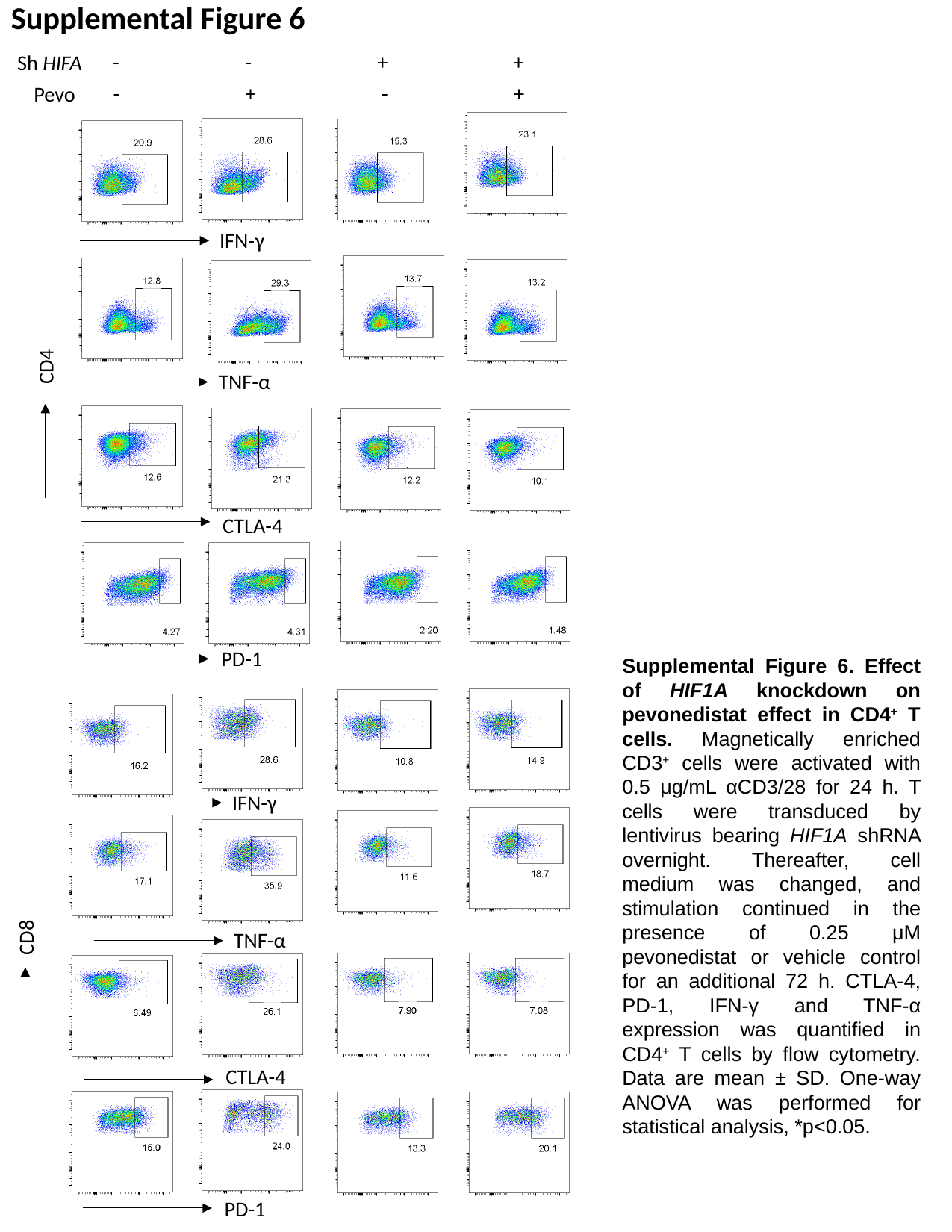

Supplemental Figure 6
 - - + +
Sh HIFA
 - + - +
Pevo
IFN-γ
CD4
TNF-α
CTLA-4
PD-1
Supplemental Figure 6. Effect of HIF1A knockdown on pevonedistat effect in CD4+ T cells. Magnetically enriched CD3+ cells were activated with 0.5 μg/mL αCD3/28 for 24 h. T cells were transduced by lentivirus bearing HIF1A shRNA overnight. Thereafter, cell medium was changed, and stimulation continued in the presence of 0.25 μM pevonedistat or vehicle control for an additional 72 h. CTLA-4, PD-1, IFN-γ and TNF-α expression was quantified in CD4+ T cells by flow cytometry. Data are mean ± SD. One-way ANOVA was performed for statistical analysis, *p<0.05.
IFN-γ
CD8
TNF-α
CTLA-4
PD-1

## Slide 7
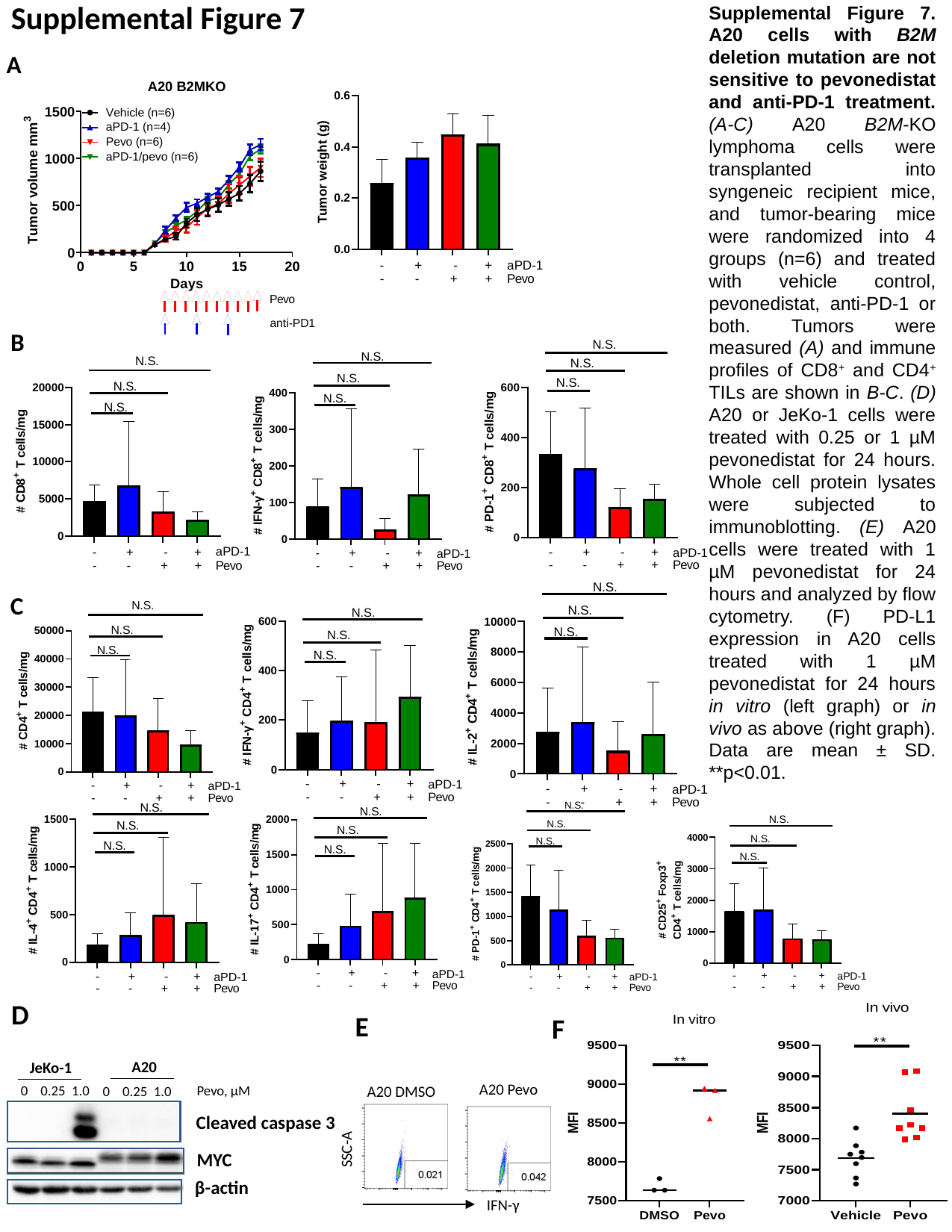

Supplemental Figure 7
Supplemental Figure 7. A20 cells with B2M deletion mutation are not sensitive to pevonedistat and anti-PD-1 treatment. (A-C) A20 B2M-KO lymphoma cells were transplanted into syngeneic recipient mice, and tumor-bearing mice were randomized into 4 groups (n=6) and treated with vehicle control, pevonedistat, anti-PD-1 or both. Tumors were measured (A) and immune profiles of CD8+ and CD4+ TILs are shown in B-C. (D) A20 or JeKo-1 cells were treated with 0.25 or 1 µM pevonedistat for 24 hours. Whole cell protein lysates were subjected to immunoblotting. (E) A20 cells were treated with 1 µM pevonedistat for 24 hours and analyzed by flow cytometry. (F) PD-L1 expression in A20 cells treated with 1 µM pevonedistat for 24 hours in vitro (left graph) or in vivo as above (right graph). Data are mean ± SD. **p<0.01.
A
B
C
D
E
F
A20
JeKo-1
A20 Pevo
A20 DMSO
Pevo, µM
0 0.25 1.0
0 0.25 1.0
Cleaved caspase 3
SSC-A
MYC
β-actin
IFN-γ

## Slide 8
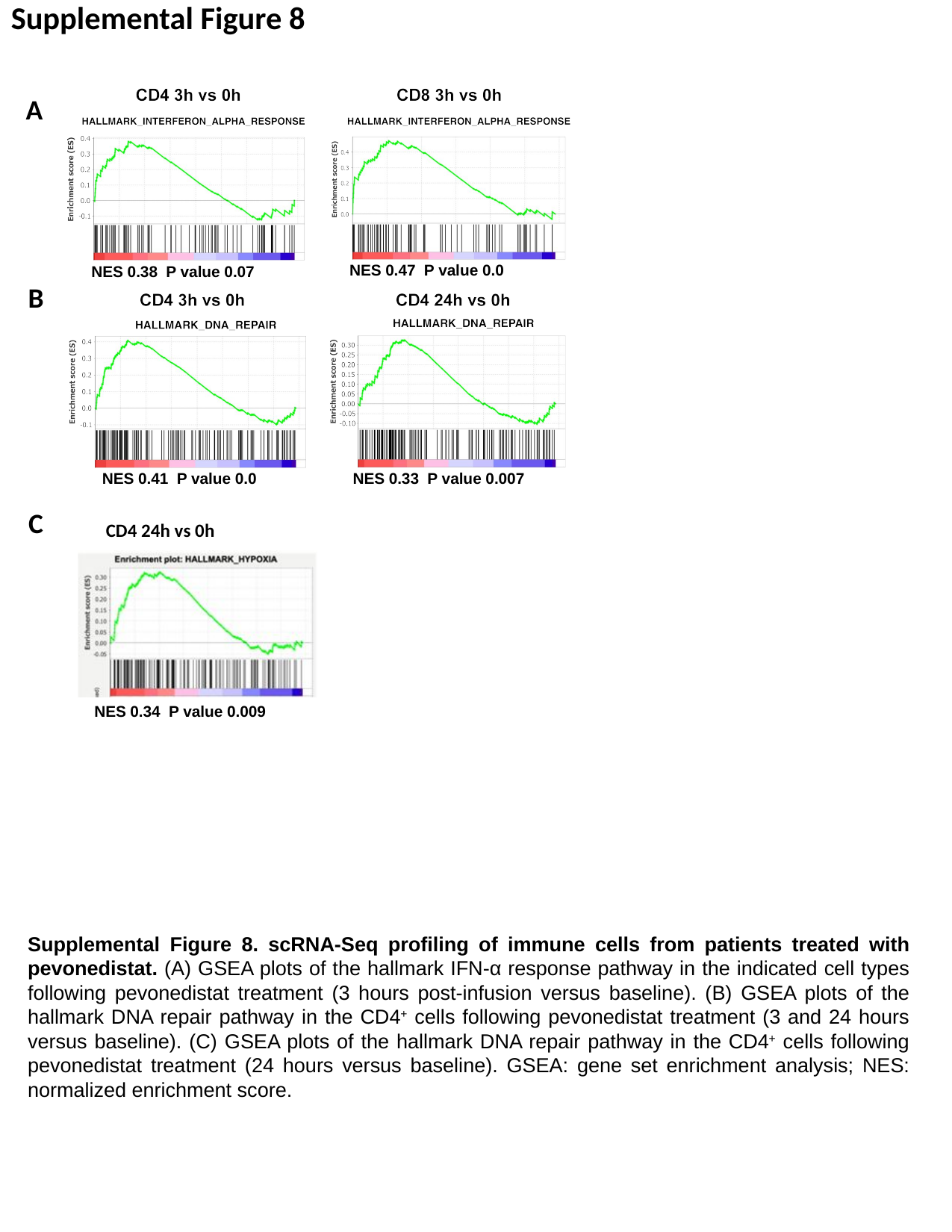

Supplemental Figure 8
A
NES 0.47 P value 0.0
NES 0.38 P value 0.07
B
NES 0.41 P value 0.0
NES 0.33 P value 0.007
C
CD4 24h vs 0h
NES 0.34 P value 0.009
Supplemental Figure 8. scRNA-Seq profiling of immune cells from patients treated with pevonedistat. (A) GSEA plots of the hallmark IFN-α response pathway in the indicated cell types following pevonedistat treatment (3 hours post-infusion versus baseline). (B) GSEA plots of the hallmark DNA repair pathway in the CD4+ cells following pevonedistat treatment (3 and 24 hours versus baseline). (C) GSEA plots of the hallmark DNA repair pathway in the CD4+ cells following pevonedistat treatment (24 hours versus baseline). GSEA: gene set enrichment analysis; NES: normalized enrichment score.

## Slide 9
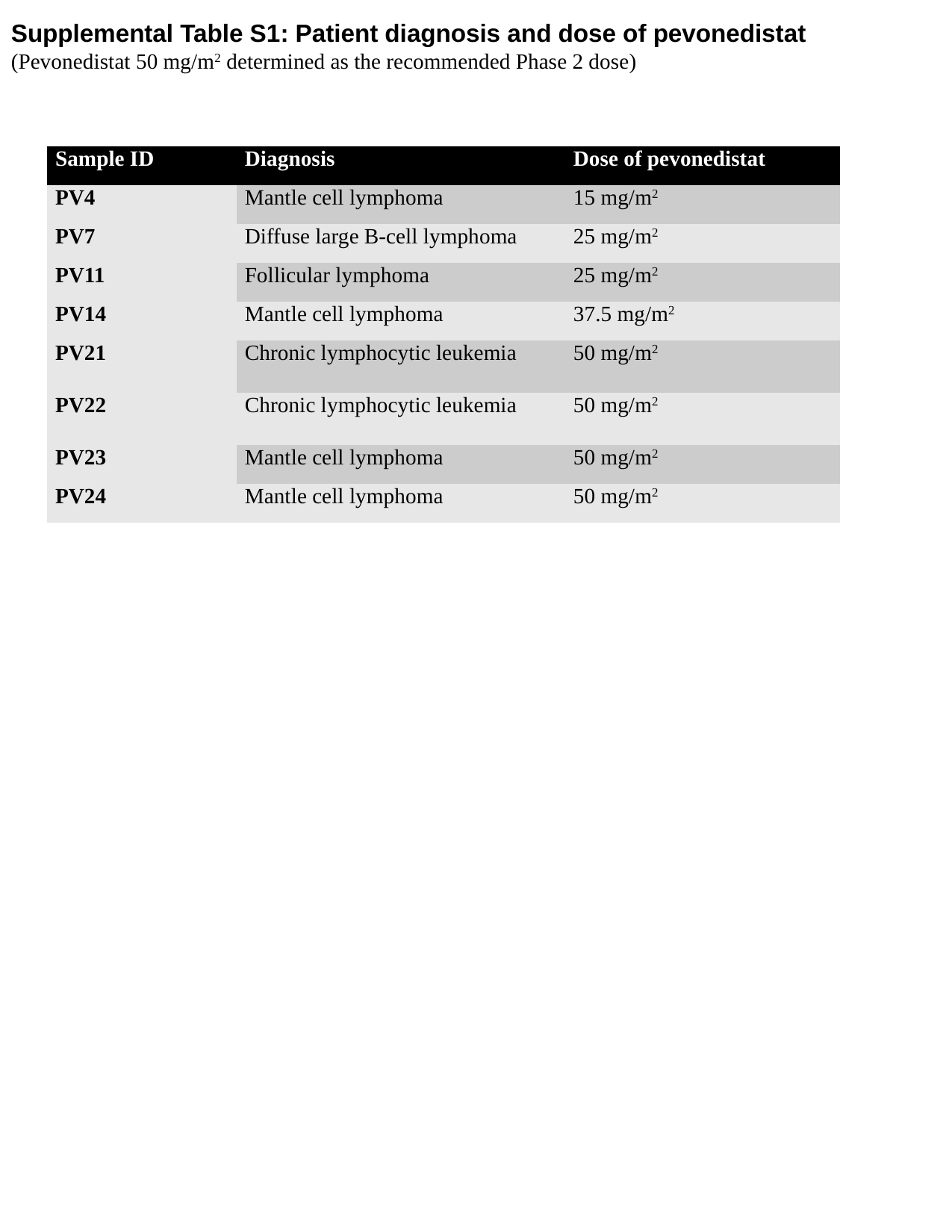

Supplemental Table S1: Patient diagnosis and dose of pevonedistat
(Pevonedistat 50 mg/m2 determined as the recommended Phase 2 dose)
| Sample ID | Diagnosis | Dose of pevonedistat |
| --- | --- | --- |
| PV4 | Mantle cell lymphoma | 15 mg/m2 |
| PV7 | Diffuse large B-cell lymphoma | 25 mg/m2 |
| PV11 | Follicular lymphoma | 25 mg/m2 |
| PV14 | Mantle cell lymphoma | 37.5 mg/m2 |
| PV21 | Chronic lymphocytic leukemia | 50 mg/m2 |
| PV22 | Chronic lymphocytic leukemia | 50 mg/m2 |
| PV23 | Mantle cell lymphoma | 50 mg/m2 |
| PV24 | Mantle cell lymphoma | 50 mg/m2 |

## Slide 10
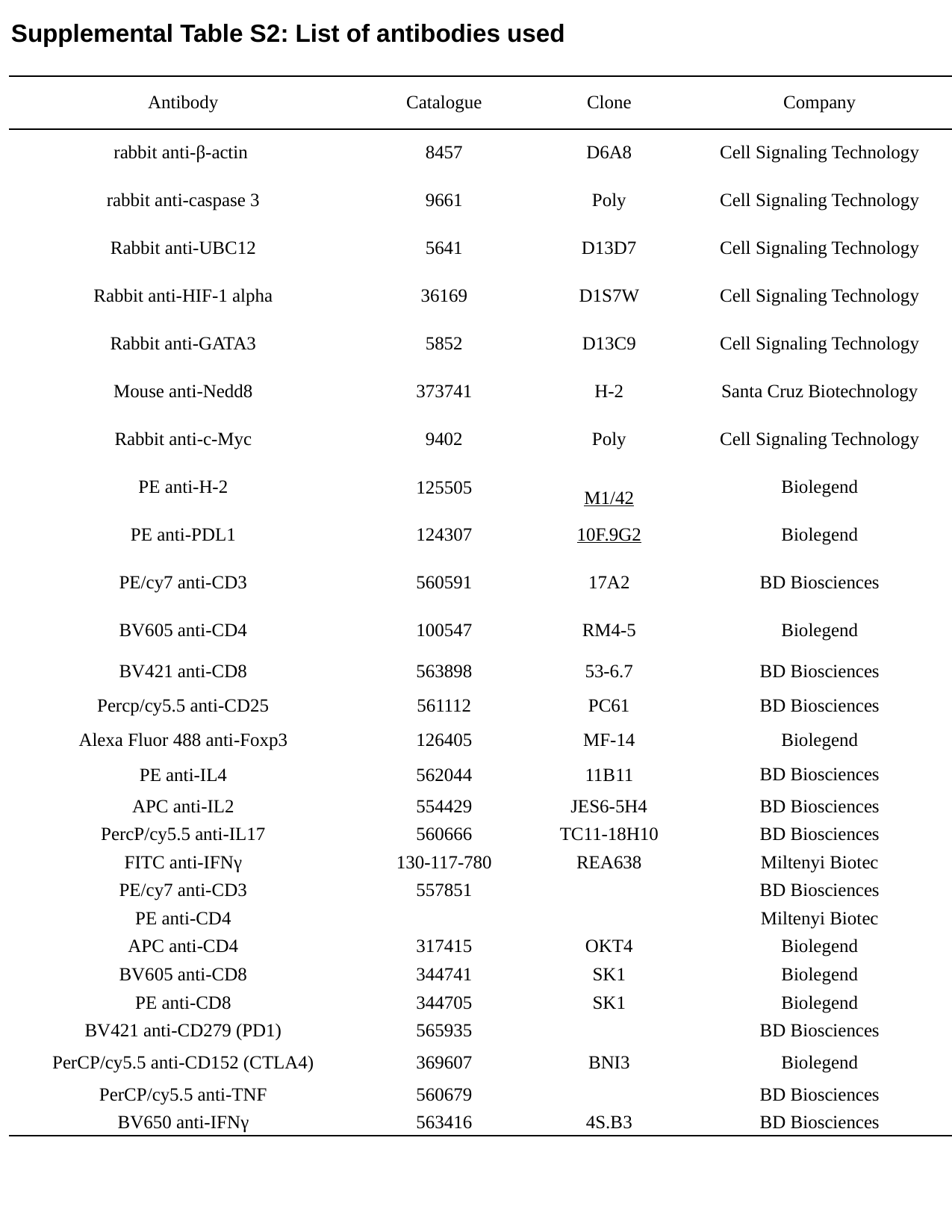

Supplemental Table S2: List of antibodies used
| Antibody | Catalogue | Clone | Company |
| --- | --- | --- | --- |
| rabbit anti-β-actin | 8457 | D6A8 | Cell Signaling Technology |
| rabbit anti-caspase 3 | 9661 | Poly | Cell Signaling Technology |
| Rabbit anti-UBC12 | 5641 | D13D7 | Cell Signaling Technology |
| Rabbit anti-HIF-1 alpha | 36169 | D1S7W | Cell Signaling Technology |
| Rabbit anti-GATA3 | 5852 | D13C9 | Cell Signaling Technology |
| Mouse anti-Nedd8 | 373741 | H-2 | Santa Cruz Biotechnology |
| Rabbit anti-c-Myc | 9402 | Poly | Cell Signaling Technology |
| PE anti-H-2 | 125505 | M1/42 | Biolegend |
| PE anti-PDL1 | 124307 | 10F.9G2 | Biolegend |
| PE/cy7 anti-CD3 | 560591 | 17A2 | BD Biosciences |
| BV605 anti-CD4 | 100547 | RM4-5 | Biolegend |
| BV421 anti-CD8 | 563898 | 53-6.7 | BD Biosciences |
| Percp/cy5.5 anti-CD25 | 561112 | PC61 | BD Biosciences |
| Alexa Fluor 488 anti-Foxp3 | 126405 | MF-14 | Biolegend |
| PE anti-IL4 | 562044 | 11B11 | BD Biosciences |
| APC anti-IL2 | 554429 | JES6-5H4 | BD Biosciences |
| PercP/cy5.5 anti-IL17 | 560666 | TC11-18H10 | BD Biosciences |
| FITC anti-IFNγ | 130-117-780 | REA638 | Miltenyi Biotec |
| PE/cy7 anti-CD3 | 557851 | | BD Biosciences |
| PE anti-CD4 | | | Miltenyi Biotec |
| APC anti-CD4 | 317415 | OKT4 | Biolegend |
| BV605 anti-CD8 | 344741 | SK1 | Biolegend |
| PE anti-CD8 | 344705 | SK1 | Biolegend |
| BV421 anti-CD279 (PD1) | 565935 | | BD Biosciences |
| PerCP/cy5.5 anti-CD152 (CTLA4) | 369607 | BNI3 | Biolegend |
| PerCP/cy5.5 anti-TNF | 560679 | | BD Biosciences |
| BV650 anti-IFNγ | 563416 | 4S.B3 | BD Biosciences |
