# Supplementary material for: Pharmacologic targeting of Nedd8-activating enzyme reinvigorates T-cell responses in lymphoid neoplasia
Source: Leukemia. 2023 Apr 8;37(6):1324–35. doi: 10.1038/s41375-023-01889-x (PMC10244170; doi:10.1038/s41375-023-01889-x)
Supplement: Supplementary file 2 — Supplementary Methods [file 41375_2023_1889_MOESM2_ESM.docx]

**Supplemental Methods**

*shRNA-mediated knockdown*

293T17 cells (ATCC) were transiently transfected with *HIF1A* shRNA (TRCN0000003808 or TRCN0000003810), *UBE2M* shRNA (TRCN0000317874) or vector control. Lentiviral particles were produced by three-plasmid transfection system using pMD2.G and psPAX2 plasmids (both from Addgene) and JetPrime transfection reagent according to the manufacturer's protocol (Polyplus-transfection), as previously described ^1^. Primary T or A20 cells were infected with viral particles by spinoculation with 1 μg polybrene and 1 M HEPES for 90 minutes at 2500 RPM and 37°C. For *HIF1A* knockdown, lentivirus containing *HIF1A* shRNA (TRCN0000003808 or TRCN0000003810) or control lentivirus were added to CD3^+^ T cells pre-stimulated by anti-CD3/CD28 antibodies for 24 hours. After 24 hours, pevonedistat was added for additional 72 hours. For *UBE2M* knockdown, lentivirus containing *UBE2M* shRNA (TRCN0000317874) or control lentivirus was added to A20 cells. After 48 hours of initial culture, puromycin selection (2 µg/mL) was performed for two weeks.

*Quantitative RT-PCR*

Cells were treated as indicated and total RNA was isolated by RNA isolation kit (Omega, USA). cDNA was synthesized from 500 ng of RNA using the qScript cDNA Supermix (QuantaBio). Quantitative real-time PCR (RT-PCR) was performed using a QuantStudio 7 Flex (Applied Biosystems) using PerfeCTaFastMix II according to the manufacturer’s instructions (QuantaBio) with template cDNA and *HIF1A* specific probe (Hs00153153). Amplification of the sequence of interest was compared to a reference probe (*GAPDH* Hs02758991, all from Thermo Fisher Scientific). All samples were analyzed in duplicate. We used the comparative Ct method for relative quantitation (2-ΔΔCt, where ΔΔCt= ΔCtP – ΔCtK; P = probe and K = reference sample).

*scRNA-seq*

*Single-cell library preparation and sequencing.* Upon thawing, PMBCs were washed and single cells were labeled with Cell Multiplexing Oligos (CMOs) (CellPlex Kit Set A, 1000261) (10X Genomics) according to the manufacturer’s instructions. Labeled samples were pooled in desired ratios. Cell concentration and viability of the pooled sample was determined using a TC20™ Automated Cell Counter (Bio-Rad). A total of 10,000 cells were targeted per pooled sample. Each sample pool was loaded into a different lane of a 10X chip (Chromium Next GEM Chip G Single Cell Kit, 1000127). cDNA libraries were generated using the Single Cell 3’ Library & Gel Bead kit version 3.1 (1000121). Indexed sequencing libraries were constructed using the reagents in the library Construction Kit (10xGenomics, 1000190). The barcode sequencing libraries were sequenced on the NovaSeq 6000 platform (Illumina) with paired-end sequencing and dual indexing. A total of 28, 10, 10 and 101 cycles were run for Read 1, i7 index, i5 index and Read 2, respectively. Raw sequences were processed and aligned to the GRCh38 genome using 10x Genomics’ CellRanger v6.1.1 “multi” pipeline with default settings, which also counted the unique molecule identifiers (UMIs) for each gene in each cell.

*scRNA-seq data processing.* The resulting gene expression matrices were loaded into R (version 4.0.3) and analyzed with the Seurat pipeline (version 4.1.0) ^2^. We required that genes had to be expressed in at least three cells to be considered for further analysis. Then, cells were filtered to retain those that contained at least 500 minimum unique genes expressed, no more than 5000 unique genes, more than 500 total UMIs and less than 10% of counts mapped to the mitochondrial genome. For the multiplexed samples from the same patient, we adopted the demultiplexing with hashtag oligos (HTOs) in the expression matrix by the function HTODemux implemented in Seurat with default parameters. Doublets were further removed using DoubletFinder (v2.0.3) with default parameters and a doublet rate threshold of 4% ^3^. For the leftover cells of 3 samples in each patient, the function “SCTransform” in Seurat was used to normalize data, scale data, and find variable features. After that, the 30 most informative principal components (PCA) of the selected top variable genes were used for cluster and visualizing all cell types. Clusters were identified by the FindClusters function with a resolution of 0.1 and visualized using the Uniform Manifold Approximation and Projection (UMAP) for dimension reduction ^4^. The cell type of each cluster was annotated by SingleR (v1.8.1) following the standard procedure using the human PrimaryCell Atlas as the reference.

*Gene Set Enrichment Analysis (GSEA) in single-cell RNA-seq.* Since we had multiple patients for each treatment condition, we employed the pseudo-bulk approach to identify the differential gene expression (DEG) patterns across different treatment time points, which is the best practice for single-cell DEG analysis to avoid inflating p-values. First, within each cell type in each patient, we aggregated the raw counts of cells of each time point into one pseudo-bulk level quantification. Then, given the multiple replicates of pseudo-bulk gene expressions of the same cell types at the three time points, we performed differential gene-expression analysis across different conditions (3 vs 0 hours, 24 vs 0 hours) by using the R package ‘DESeq2’ (v1.36.0), which generated a pre-ranked gene list as the input for the GSEA analysis to quantify the relative enrichment of gene sets affected by the treatment in each cell type. The count matrix was initially filtered to include only protein-coding genes, with a mean of >1 read per sample. To determine whether genes upregulated in pevonedistat versus baseline were enriched in clusters, GSEA pre-ranked tool was used to perform GSEA software (version 4.2.3)^5^. with the MSigDB hallmark gene sets (v7.2) computed and following parameters: number of permutations = 2000, min size (exclude smaller sets) = 20, collapsing mode for probes sets (=> 1 gene) = max_probe, plot graphs for the top sets of each phenotype = 100, and others set to default.

1. Hashiguchi T, Bruss N, Best S, Lam V, Danilova O, Paiva CJ*, et al.* Cyclin-Dependent Kinase-9 Is a Therapeutic Target in MYC-Expressing Diffuse Large B-Cell Lymphoma. *Molecular Cancer Therapeutics* 2019 Sep; **18**(9)**:** 1520-1532.

2. Hao Y, Hao S, Andersen-Nissen E, Mauck WM, 3rd, Zheng S, Butler A*, et al.* Integrated analysis of multimodal single-cell data. *Cell* 2021 Jun 24; **184**(13)**:** 3573-3587 e3529.

3. McGinnis CS, Murrow LM, Gartner ZJ. DoubletFinder: Doublet Detection in Single-Cell RNA Sequencing Data Using Artificial Nearest Neighbors. *Cell Syst* 2019 Apr 24; **8**(4)**:** 329-337 e324.

4. Becht E, McInnes L, Healy J, Dutertre CA, Kwok IWH, Ng LG*, et al.* Dimensionality reduction for visualizing single-cell data using UMAP. *Nat Biotechnol* 2018 Dec 3.

5. Subramanian A, Tamayo P, Mootha VK, Mukherjee S, Ebert BL, Gillette MA*, et al.* Gene set enrichment analysis: a knowledge-based approach for interpreting genome-wide expression profiles. *Proc Natl Acad Sci U S A* 2005 Oct 25; **102**(43)**:** 15545-15550.
